# Bioenergetic Mechanisms of Seizure Control

**DOI:** 10.3389/fncel.2018.00335

**Published:** 2018-10-08

**Authors:** Richard Kovács, Zoltan Gerevich, Alon Friedman, Jakub Otáhal, Ofer Prager, Siegrun Gabriel, Nikolaus Berndt

**Affiliations:** ^1^Charité – Universitätsmedizin Berlin, Corporate Member of Freie Universität Berlin, Humboldt-Universität zu Berlin, and Berlin Institute of Health, Institut für Neurophysiologie, Berlin, Germany; ^2^Departments of Physiology and Cell Biology, Cognitive and Brain Sciences, The Zlotowski Center for Neuroscience, Ben-Gurion University of the Negev, Beersheba, Israel; ^3^Department of Medical Neuroscience, Faculty of Medicine, Dalhousie University, Halifax, NS, Canada; ^4^Institute of Physiology, Czech Academy of Sciences, Prague, Czechia; ^5^Charité – Universitätsmedizin Berlin, Corporate Member of Freie Universität Berlin, Humboldt-Universität zu Berlin, and Berlin Institute of Health, Institut für Biochemie, Berlin, Germany; ^6^Charité – Universitätsmedizin Berlin, Corporate Member of Freie Universität Berlin, Humboldt-Universität zu Berlin, and Berlin Institute of Health, Institute for Computational and Imaging Science in Cardiovascular Medicine, Berlin, Germany

**Keywords:** seizure, lactate, adenosine, neurometabolic coupling, neurovascular coupling, pericyte

## Abstract

Epilepsy is characterized by the regular occurrence of seizures, which follow a stereotypical sequence of alterations in the electroencephalogram. Seizures are typically a self limiting phenomenon, concluding finally in the cessation of hypersynchronous activity and followed by a state of decreased neuronal excitability which might underlie the cognitive and psychological symptoms the patients experience in the wake of seizures. Many efforts have been devoted to understand how seizures spontaneously stop in hope to exploit this knowledge in anticonvulsant or neuroprotective therapies. Besides the alterations in ion-channels, transmitters and neuromodulators, the successive build up of disturbances in energy metabolism have been suggested as a mechanism for seizure termination. Energy metabolism and substrate supply of the brain are tightly regulated by different mechanisms called neurometabolic and neurovascular coupling. Here we summarize the current knowledge whether these mechanisms are sufficient to cover the energy demand of hypersynchronous activity and whether a mismatch between energy need and supply could contribute to seizure control.

## Introduction

Epilepsy is a common neurological disease characterized by the manifestation of unprovoked seizures (Fisher et al., 2014). An epileptic seizure is “a transient occurrence of signs and/or symptoms due to abnormal excessive or synchronous neuronal activity in the brain” (Fisher et al., 2005). Seizures are represented by a stereotypic sequence of electroencephalographic events and associated clinical symptoms. Seizure termination can be described as a homeostatic self-limitation of the hypersynchronous activity, representing not a mere cessation but a characteristic change in the excitability of the nervous tissue. These changes evolve successively and may already start during the course of the seizure before the activity would terminate (Essig and Flanary, 1966). The current review focus on the contribution of altered energy metabolism to seizure control. For reviews on other anticonvulsant mechanisms mediated by intrinsic ion-channel modifications, changes in functional, network and synaptic properties of the neurons and effects of neuromodulators see Löscher and Köhling (2010) and Zubler et al. (2014).

Seizures represent an extraordinary burden on the energy metabolism of the brain as restoration of the transmembrane ion gradients is mediated by energy consuming pump mechanisms. Thus, it is tempting to speculate that the metabolic changes that develop slowly during the course of a seizure might finally lead to the cessation of the hypersynchronous activity. Metabolic alterations may include altered substrate and oxygen availability, accumulation of metabolic intermediates and byproducts (such as adenosine, lactate and CO_2_) which in turn leads to activation of adenosine receptors, extracellular acidosis and opening of K_ATP_ channels due to local shortage of ATP. These changes build up consecutively during the course of a seizure establishing conditions that not only contribute to the cessation of the activity but could potentially alter the normal neuronal functioning in the postictal phase. Conversely, disturbed energy metabolism could by itself favor seizure initiation by altering the ability of the tissue to recover transmembrane ion gradients following neuronal activity, as it is seen in mitochondrial encephalopathies (Kang et al., 2013; Zsurka and Kunz, 2015) or in mesial temporal lobe epilepsy (Rowley and Patel, 2013; Boison and Steinhäuser, 2018). Interictal hypoperfusion (Guillon et al., 1998; Suh et al., 2005; Geneslaw et al., 2011) and hypometabolism (Hetherington et al., 1995; Cendes et al., 1997; Hong et al., 2002) have been demonstrated in a wide range of epileptic syndromes including temporal lobe epilepsy, generalized childhood absence epilepsy and status epilepticus. Although seizure-induced cell loss and sclerotic modification of the tissue could partially explain the hypometabolic state, there is also evidence for dysfunction of the neurometabolic coupling (Kann et al., 2005) and oxidative damage of respiratory enzymes (Kunz et al., 2000; Vielhaber et al., 2003; Folbergrová and Kunz, 2012; Rowley and Patel, 2013). Lasting deficiencies in energy metabolism might underlie the frequent observation that “seizures-beget-seizures” (Ben-Ari, 2001; Farrell et al., 2017b) by favoring the initiation of subsequent hypersynchronous activity (Zsurka and Kunz, 2015). Mismatch in the neurometabolic coupling might be responsible for seizure-associated cell loss via either free radical dependent or hypoxic mechanisms (Ingvar, 1986; Frantseva et al., 2000; Kovács et al., 2002; Rowley and Patel, 2013). Metabolic disturbances might also contribute to the development of pharmacoresistance, i.e., the incapability to prevent seizures with two or more antiepileptic therapy regimens directly or indirectly following sclerotic transformation of the tissue (Kovács and Heinemann, 2014). Thus, it is fundamental to understand the potentially pro- or anticonvulsant consequences of the seizure-associated changes in energy metabolism.

In the following we provide a short overview of the literature about the alterations of neurometabolic coupling during seizures and how these could contribute to seizure control. We discuss possible therapeutic targets which exploit metabolism-related mechanisms (2-deoxy-D-glucose (2DG), pyruvate, fructose 1,6 bisphosphate, ketogenic diet, CO_2_ inhalation, carbonic anhydrase inhibitors, adenosine 1 agonists). The last part is devoted to the mechanisms of seizure-induced neurovascular decoupling, which might be responsible for postictal hypometabolism and have been suggested to be associated with cognitive disturbances. We apologize that we cannot extensively discuss all aspects of seizure termination and regulation of neuronal energy metabolism as well as the anti-seizure mechanisms associated to caloric restriction and ketogenic diet.

## Neurometabolic Coupling During Acute Seizures and in Chronic Epileptic Tissue

**FIGURE 1:**
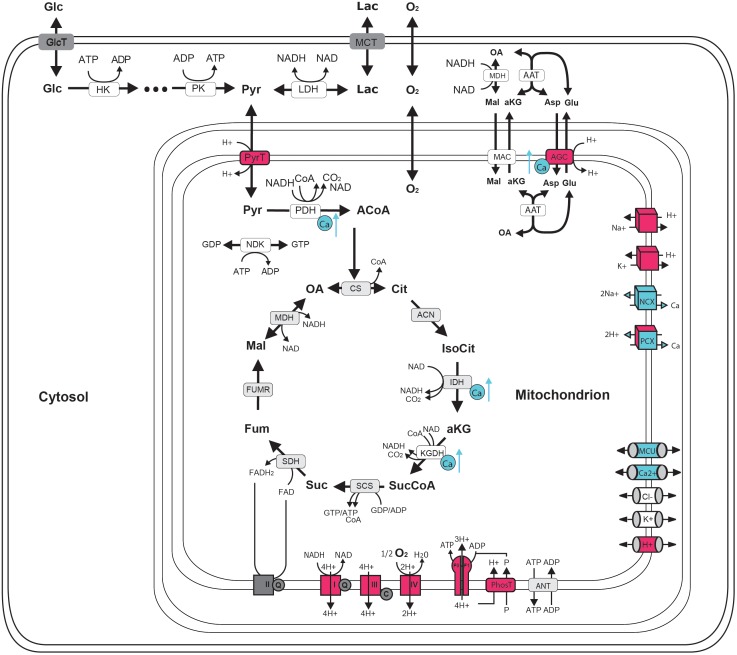
Schematic representation of central neuronal energy metabolism (adapted from Bedner et al., 2015). Proton dependent processes are presented in magenta and calcium dependent processes are presented in blue. Glycolysis degrades glucose beginning with hexokinase (hk) dependent phosphorylation to glucose-6-phosphate ending with pyruvate kinase (pk) dependent formation of pyruvate. Pyruvate can by either transformed to lactate by the lactate dehydrogenase (LDH) and be exported from the cell by monocarboxylate transporter (MCT) (anaerobic glycolysis) or taken up into the mitochondria via proton symport by pyruvate transporter (PyrT) with subsequent conversion to Acetyl-Coa by pyruvate dehydrogenase (PDH) for oxidation to CO_2_ in the citric acid cycle (CAC) (aerobic glycolysis). The CAC cycle, starting with the formation of citrate (Cit) from oxaloacetate (OA) and acetyl-CoA (ACoA) by citrate synthase (CS) and proceeding via the formation of isocitrate (IsoCit) by aconitase (ACN), α-ketoglutarate (aKG) by isocitrate dehydrogenase (IDH), succinyl-CoA (SucCoA) by α-ketoglutarate dehydrogenase, succinate (Suc) by succinyl-coA synthetase, fumarate (Fum) by succinyldehydrogenase, malate (Mal) by fumerase (FUMR) and the replenishing of OA by malate dehydrogenase (MDH), forms NADH and FADH2 used by the respiratory chain (RC). Complex I-IV of the RC are proton pumps generating the proton motive force that determine the rate of proton-assisted ion transport of Na+, K+, Ca2+ and phosphate (P) across the inner mitochondrial membrane, the rate of the adenine nucleotide transporter (ANT) exchanging mitochondrial ATP against cytosolic ADP and the rate of ATP synthesis by the F0F1-ATPase (F0F1). Shuttling of electrons (NAD-bound hydrogen) between the cytosol and the mitochondrial matrix is catalyzed by the Malate-Asparte shuttle, comprising the malate/α-ketoglutarate carrier (MAC), the mitochondrial and cytosolic aspartate amino transferase (AAT) and the proton driven antiport of glutamate (Glu) and aspartate (Asp) by the aspartate/glutamate carrier (AGC). Besides protons, calcium plays a key role in neuronal energy metabolism as it controls the key mitochondrial dehydrogenase PDH, IDH and KGDH and the rate of electron shuttling by AGC (ARALAR). Calcium uptake into the mitochondrion proceeds in a calcium dependent manner by MCU thereby coupling neuronal excitation with neuronal energy metabolism.

Neuronal activity results in local changes in transmembrane ion-gradients, such as the increase in extracellular [K^+^] which is immediately countered by a concurrent increase in Na-K-ATPase activity (Lux et al., 1986). In order to keep pace with the ATP-demand of the ion-pump activity as well as that of the transport processes related to synaptic signaling (Liotta et al., 2012; Hall et al., 2012), energy metabolism has to adopt to the different neuronal activity states (Ames, 2000; Heinemann et al., 2002; Berndt et al., 2015). This neurometabolic coupling could operate via “pull” and “push” mechanisms, i.e., either as a consequence of alterations in ATP or other energy metabolism intermediates, or in a feed forward manner by activity dependent changes in intracellular and intramitochondrial Ca^2+^, K^+^ and nitric oxide (NO) concentrations (Szewczyk et al., 2006; Denton, 2009). During physiological and interictal activity, changes in overall intracellular ATP concentration are expected to be rather small (see Seizure Associated Alterations in Energy Metabolism Intermediates and pH). Yet, an increase in ATP utilization by the Na-K-ATPase leads to a concomitant increase in ADP and AMP concentration, as the adenylate kinase maintains a fast equilibrium between the adenine nucleotides. Assuming an ATP level of 3mM and an ADP level of 0.3 mM, even a minor decrease in ATP by 10% would decrease the ATP/ADP ratio from 10 to 4.5 and increase AMP levels more than fourfold. These changes in adenylates have immediate consequences for the regulation of energy metabolism (**Figure 1**). First, the adenine nucleotide translocator is activated leading to a decrease in mitochondrial ATP and mitochondrial membrane potential (ψ) as the exchange of ATP (3 negative charges) for ADP (2 negative charges) is an electrogenic process. The shift in cytosolic and mitochondrial ATP/ADP ratio and the decrease in ψ activate F0F1-ATPase, further draining the ψ. As a consequence, complexes IV, III, and I of the ETC are activated by associated shifts in the electron carriers, cytochrome C and ubiquinone (QH_2_). This activation results in the initial oxidation shift in mitochondrial NADH, which can be observed by NADH-autofluorescence (Kovács et al., 2001; Schuchmann et al., 2001; Berndt et al., 2015). The decrease in mitochondrial NADH activates the regulatory dehydrogenases of the tricarboxylic acid cycle as well as the pyruvate dehydrogenase reaction, thereby channeling pyruvate into the tricarboxylic acid cycle. At the same time, the glycolytic pathway is activated by the decreased ATP/ADP ratio and the increase in AMP at the glucokinase, phosphofructokinase and pyruvate kinase level, providing the pyruvate needed for increased aerobic metabolism. Normally, the increase in glycolytic activity exceeds the increase in aerobic metabolism and the excess pyruvate is exported in the form of lactate (Berndt et al., 2015).

The “push” mechanisms, such as the mitochondrial Ca^2+^-cycle, are complementary in regulating energy metabolism (Kovács et al., 2005; Wei et al., 2011; Berndt et al., 2015; Nicholls, 2017; Kannurpatti, 2017). Neuronal firing and synaptic activity results in localized transient cytoplasmic Ca^2+^ increases, which are sequestered by mitochondria via the mitochondrial Ca^2+^ uniporter (MCU) channel complex leading to increased matrix Ca^2+^ concentration. Matrix Ca^2+^ plays a key regulatory role as it can enhance ETC in a potassium dependent manner (Szewczyk et al., 2006), increase aspartate malate shuttle and pyruvate supply (Gellerich et al., 2012; Rueda et al., 2014) and it is a positive modulator of three key dehydrogenases of the tricarboxylic acid cycle (TCA, McCormack et al., 1990; Denton, 2009), thereby leading to the enhanced formation of reducing equivalents (**Figure 1**). Typical biphasic changes in the redox state of flavin and adenine dinucleotides (FADH_2_ and NADH) can be observed following physiological stimulus *in vivo* (Kasischke et al., 2004) and during seizure-like events in brain slices consisting of an initial oxidation and a lasting reduction which outlasts the hypersynchronous activity (Schuchmann et al., 1999; Kovács et al., 2001). In this latter preparation enhanced Ca^2+^ cycling across the mitochondrial membrane during seizures has been shown to be responsible for the dissipation of ψ (Kovács et al., 2005), although the contribution of the mitochondrial permeability transition induced by high mitochondrial Ca^2+^-load cannot be completely excluded (Kovac et al., 2012). While mitochondrial depolarization by definition decreases the proton motive force (composed of Δψ and ΔpH ), it could also represent one way to enhance respiration (Malinska et al., 2010). Due to the fixed stoichiometry of the F0F1-ATPase of three protons per generation of one ATP, the decrease in proton motive force will have no energetic efficiency consequences as long as the energy of the three protons exceed the formation enthalpy of ATP. However, the ψ dependence of the F0F1-ATPase activity leads to a strong activation and thereby increased ATP formation (see above). Thus, seizure termination *in vitro* seems to occur in the presence of enhanced energy metabolism, when neither the availability of the reducing equivalents nor oxygen could represent a limitation for the electrical activity (Schoknecht et al., 2017). However, in case of frequently recurring seizure-like events, which would resemble status epilepticus *in vivo*, signs of metabolic impairment develop in a free radical dependent manner (Kovács et al., 2002; Malinska et al., 2010; Zsurka and Kunz, 2015). Under these conditions seizure termination is impaired and the electrical activity transforms from seizure-like events to pharmacoresistant late recurrent discharges, indicating that the slowly developing energy crisis is rather pro-epileptic (Schuchmann et al., 1999; Ivanov et al., 2015). Seizure-associated enhancement of mitochondrial free radical formation might play an important role in the development of metabolic disturbances both *in vitro* (Frantseva et al., 2000; Kovács et al., 2002) and *in vivo* (Kunz et al., 2000; Vielhaber et al., 2003; Rowley and Patel, 2013). In addition, the seizure associated increase in NO formation was found primarily pro-epileptic as blockade of the neuronal NO synthase decreased severity of kainic acid induced seizures *in vivo* (Beamer et al., 2012) and delayed seizure onset *in vitro* (Schuchmann et al., 2002; Kovács et al., 2009). Unfortunately, it is not possible to unequivocally determine the contribution of NO-mediated inhibition of the electron transport chain complexes to the pro-epileptogenic effect (Ledo et al., 2010), as NO influences neuronal excitability and cerebral blood flow in many different ways.

**FIGURE 2:**
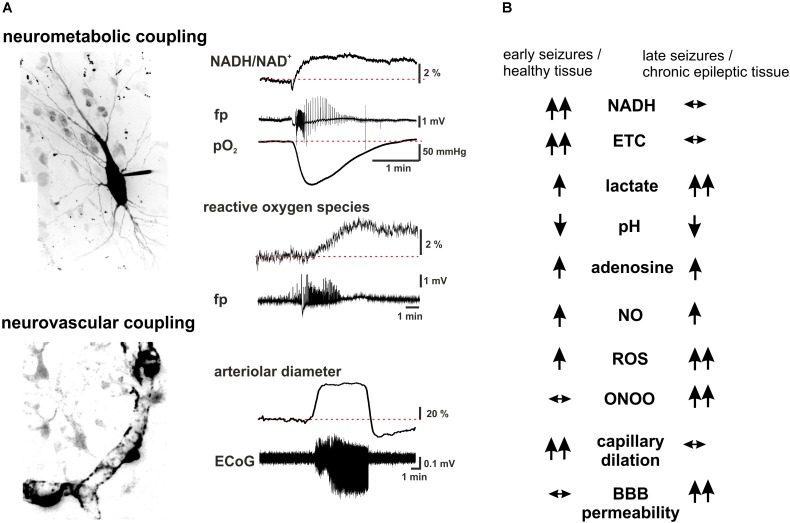
Summary scheme of seizure-associated changes in cellular energy metabolism and cerebral blood. **(A)** Seizure-associated changes in transmembrane ion gradients ultimately activate neuronal energy metabolism in order to counterbalance the ATP-need of the ion pump activity. In brain slices kept under constant carbogen gassing conditions seizure-associated enhancement of respiration can be measured as a drop in pO_2_ whereas a shift in the fluorescence of NADH represents the metabolism-dependent redox shift of this electron carrier (fp: local field potential). Whereas the respiration slowly returns to the pre-seizure values following cessation of the activity, the reducing shift in the NADH/NAD^+^ ratio outlast the changes in respiration. This mismatch might favor the late enhancement of reactive oxygen species (ROS) formation, as represented here by the oxidation of the mitochondrially targeted ethidium derivative, MitoSox in the same preparation. Seizures are associated with reversible arteriolar and capillary vasodilation, the latter likely mediated by contractile pericytes, and a consequent overshooting pO_2_ response. **(B)** There are considerable differences in the seizure-associated neurometabolic and neurovascular responses between early and late events during status epilepticus, as well as in healthy and chronic epileptic tissue (arrows indicate the direction of the change of individual parameters). Seizure-associated overshooting NADH reduction eroded during recurrent seizures *in vitro* and it was almost absent in chronic epileptic tissue from epilepsy patients, due to the free radical dependent damage of mitochondrial dehydrogenases. On the other hand, mitochondrial injury might in turn enhance subsequent formation of reactive oxygen species (ROS) in a vicious cycle. Seizures resulted in lactate release both in control and chronic epileptic tissue samples, whereas in the latter even interictal lactate levels were increased likely reflecting altered expression pattern of monocarboxylate transporters and energy metabolism of glial cells. Seizure-associated enhancement of respiration and lactate release result in a metabolic acidosis, which – along with the enhanced adenosine levels- represents a strong anticonvulsant mechanisms even in chronic epileptic tissue. Despite the induction of massive CBF increase by seizures vascular responsiveness was found to be decreased following status epilepticus, and changes in pericytic reactivity are observable already few minutes after seizure onset. This could be brought by oxidative stress of these cells as described in the no reflow phenomenon after hypoxia-reperfusion. Although seizure-associated increase in nitric oxide (NO) formation contributes to the hyperemia, in the presence of superoxide NO is turned into peroxynitrite with deleterious consequences on microcirculation. Postictal hyporesponsiveness of the microvasculature has been speculated to underlie cognitive disturbances and epilepsia comorbidities, whereas damage of the blood-brain barrier (BBB), albumin leakage and the subsequent transformation of the nervous tissue were shown to be pro- epileptogenic. (hippocampal pyramidal cell and capillary pericytes are just for illustration purposes, some of the traces in A are adopted from Malinska et al., 2010).

Most of the aforementioned findings were obtained in experiments with acutely induced epileptiform activity in tissue samples of otherwise healthy animals. However, in chronic epileptic tissue the metabolic consequences of prior activity might influence the effects of seizures on energy metabolism. Indeed, signs of metabolic impairment were evident in sclerotic hippocampal tissue from epilepsy patients as well as in the pilocarpine model of epilepsy, which may correspond to the hypoperfusion and hypometabolism in these areas (Kunz et al., 2000; Vielhaber et al., 2003; Kann et al., 2005). In conclusion, although metabolism related factors (see below) might contribute to seizure termination, this can also happen in the presence of intact neurometabolic coupling and - at least *in vitro*- no obvious restriction of glucose and oxygen supply (**Figure 2**). On the other hand, metabolic disturbances during frequently recurring seizures or in chronic epileptic tissue rather favor than limit the occurrence of hypersynchronous activity (Otáhal et al., 2014, and citations therein).

## Seizure Associated Alterations in Energy Metabolism Intermediates and pH

Recurrent seizures represent an extraordinary metabolic burden, leading to increased tissue lactate and decreased phosphocreatine, glucose and glycogen content (Folbergrová et al., 2000). One plausible explanation for self-limitation of the hypersynchronous activity would be an exhaustion of the high energy phosphate and/or the glucose reserves. Neuronal metabolism is fueled almost exclusively by glucose as substrate, but the role of glucose availability in seizure development and termination is controversial. The glucose transporter GLUT1 plays an essential role in capillary to brain glucose transport and mutations in the SLC2A1 gene that encodes GLUT1 lead to deficiency of the transporter and cause several types of generalized epilepsy (Hildebrand et al., 2014). Epilepsy patients with diagnosed GLUT1 deficiency highly benefit from ketogenic diet. Severe hypoglycaemia by itself is known to induce seizures and moderate hypoglycemia decreased the threshold of seizure induction by fluorothyl *in vivo* (Kirchner et al., 2006). This could be brought about by the enhanced excitability as a consequence of altered Na-K-ATPase activity. Alternatively, an imbalance of excitatory and inhibitory neuron tonus has been also suggested, as the latter cells are more dependent on oxidative metabolism (Pumain et al., 2008). On the contrary, hypoglycaemia exerted anticonvulsant effects *in vitro* on low-Mg^2+^ induced seizures (Kirchner et al., 2006). With respect to another immediate energy resource, phosphocreatine, it was shown that brain-type creatine kinase deficient transgenic mice were less prone to develop seizures under pentylenetetrazole. In these animals initial discharges developed into a depression, which suggests that an energy crisis might actually contribute to seizure termination (Streijger et al., 2010). However, the evidence for an energy crisis in terms of ATP depletion is rather limited in other animal models of epilepsy. ATP and high energy phosphate levels are kept close to normal even during recurrent seizures (status epilepticus) lasting for hours as was shown in an NMR study of kainate-induced status (Meric et al., 1994). By modeling seizure associated changes in energy metabolism intermediates and cerebral metabolic rate of oxygen consumption (CMRO_2_) in a brain slice preparation Schoknecht and colleges showed that a fivefold increase in CMRO_2_ was able to compensate for the enhanced energy demand of seizures whereas the decrease in overall cytosolic ATP content remained relatively small (Schoknecht et al., 2017). Nevertheless, in certain metabolic compartments – such as the axonal/presynaptic domain of fast spiking interneurons - larger local ATP consumption could be hypothesized (Hu et al., 2018).

In addition to the increased blood flow and the consequent increase in glucose supply, astrocytic glycogen reserves are also mobilized during seizures (Folbergrová et al., 2000). Astrocytes form an electrically and metabolically coupled network of cells (Boison and Steinhäuser, 2018), which is capable to transport glucose to the most active areas during seizure-like events *in vitro* (Rouach et al., 2008). Impairment of gap junctional coupling and connexin expression of astrocytes is often observed in chronic epileptic tissue (Bedner et al., 2015). Whether these changes contribute to the control of seizures remains controversial as both, pro- and anticonvulsant effects of the uncoupled network are possible, via impaired potassium, glutamate buffering and nutrient redistribution, respectively (Boison and Steinhäuser, 2018; and citations therein).

In addition to enhanced oxidative metabolism, surplus glucose can be turned into lactate by anaerob metabolism. Interstitial lactate accumulation during seizures is a general finding in patients, animals as well as in *in vitro* models of epilepsy (Calabrese et al., 1991; Meric et al., 1994; Doğan et al., 2017). In epilepsy patients interictal lactate levels were found to be elevated, suggesting altered energy metabolism and lactate turnover in general (Cavus et al., 2005). Although lactate is effectively removed into the bloodstream, it has been long speculated that lactate might serve as an alternative substrate for energy metabolism of neurons under conditions of increased demand. Despite the controversy on the validity of astrocytic-neuronal lactate shuttle (ANLS) (Pellerin and Magistretti, 2012; Dienel, 2017), lactate seems to play a role in synaptic transmission and plasticity in the presence of ample glucose and might support energy demanding network oscillations in the gamma range (Galow et al., 2014; Nagase et al., 2014). Monocarboxylate transporters (MCT) mediate lactate uptake and release, depending on the transporter affinity and the concentration gradients, although lactate release via ion channels has been also described (Pierre and Pellerin, 2005; Sotelo-Hitschfeld et al., 2015). MCT4 is the major MCT isoform present with particularly high density at the astrocytic endfeet enwrapping the cerebral microvasculature (Pierre and Pellerin, 2005), hinting that MCT4 facilitates the removal of lactate into the circulation. In addition to the perivascular endfeet, the high affinity MCT2 isoform is preferentially localized on post-synaptic structures (Bergersen et al., 2005; Bergersen, 2007) suggesting that this isoform might be responsible for the metabolic support of synaptic signaling (Galeffi et al., 2007; Nagase et al., 2014). The low affinity isoform MCT1 is expressed in astrocytes, oligodendrocytes and capillary endothelial cells and it is critically involved in oligodendrocyte development and maintenance of axonal functions (Lee et al., 2012).

We have recently shown that intrinsic lactate supports synaptic signaling in rat hippocampal slices and it contributes to energy demand of the restoration of transmembrane ion gradients (Angamo et al., 2016). Inhibition of the MCT2-mediated lactate transport by α-cyano-4-hydroxycinammic acid (4CIN) led to a decrease in postsynaptic action potential generation and this effect was dependent on the activation of K_ATP_ channels, indicative of local energy shortage. Indeed, MCT inhibition reduced basal and stimulus-dependent CMRO_2_ and induced marked alterations in the redox state of mitochondrial FADH_2_ (Angamo et al., 2016). With respect to hypersynchronous activity we found a strong inhibition of interictal- and seizure-like events in presence of 4CIN in different *in vitro* models of epilepsy. Interestingly, while K_ATP_ channels contributed to the suppression of synaptic activity, the anticonvulsant effect of MCT inhibitors could not be blocked by glibenclamide, which is in line with the assumption that seizure-associated overall increase in ATP consumption is counterbalanced by the enhanced oxidative metabolism (Schoknecht et al., 2017). The anticonvulsant effect of MCT inhibitors was present both, in brain slices from control rats and also in chronic epileptic tissue from epilepsy surgery. This suggests that lactate support of energy metabolism during seizures is a general phenomenon and it is not affected by the observed changes in MCT expression in chronic epileptic tissue (Lauritzen et al., 2012; Angamo et al., 2017). The anticonvulsant effect of lactate uptake inhibitors complements the study of Sada and colleges, demonstrating that inhibition of the lactate dehydrogenase (LDH) with a stiripentol analog suppressed seizures in the pilocarpine model *in vivo* (Sada et al., 2015).

The suppression of seizure-like activity by MCT inhibitors was not mediated by extracellular lactate accumulation and subsequent acidosis but rather by activation of adenosine receptors following adenosine release upon enhanced neuronal activity in the presence of substrate restriction. Application of the adenosine 1 (A1) receptor antagonist DPCPX could reverse completely the antiseizure effect of 4CIN (Angamo et al., 2017) indicating the critical role of these receptors in seizure termination. Indeed, endogenous adenosine is known to be released during seizures and A1 receptors are mainly responsible for the control of neuronal excitability by adenosine (Dunwiddie, 1980; Fedele et al., 2006; Lovatt et al., 2012). Microdialysis studies in patients with temporal lobe epilepsy have also shown that adenosine levels rise as a consequence of seizures and the subsequent seizure termination is mediated by the activation of A1 receptors (During and Spencer, 1992; Van Gompel et al., 2014). A1 receptors are Gi/o protein coupled receptors which reduce the release of glutamate by inhibiting voltage-gated calcium channels (Kuroda, 1978; Mogul et al., 1993) and decrease postsynaptic excitability of neurons by enhancing G-protein coupled inwardly rectifying potassium conductances (Haas and Greene, 1984; Trussell and Jackson, 1987). Adenosine is thought to act as a neuromodulator involved in the homeostatic balance between inhibition and excitation, whereby A1 receptors may be responsible for reduced excitability and synaptic transmission subsequently resulting in a dampened network activity (Cunha, 2001; Dias et al., 2013). In fact, A1 receptors were found to decrease the power of gamma oscillations in hippocampal brain slices (Pietersen et al., 2009; Schulz et al., 2012). Activation of A1 receptors has in general a strong anticonvulsant efficacy in both rodent and human brain slices (Szybala et al., 2009; Klaft et al., 2012, 2016). A1 agonists were even capable to prevent the transition to status epilepticus-like recurrent discharges *in vitro* (Avsar and Empson, 2004) suggesting that status epilepticus may result from impaired adenosine receptor-dependent seizure termination mechanisms (Young and Dragunow, 1994; Kochanek et al., 2006; Hamil et al., 2012).

Notably, inhibiting glycolysis by application of 2DG is able to arrest seizures in different *in vivo* models (Stafstrom et al., 2008, 2009; Shao and Stafstrom, 2017). However, a more intricated set of effects is hinted by the findings that acute exposure to 2DG could either elevate or decrease the seizure threshold, depending on the epilepsy model (6 Hz stimulation versus kainic acid, PTZ, electroshock and amygdala kindling induced seizures; Gasior et al., 2010), whereas chronic application of 2DG initiated epileptogenesis (Samokhina et al., 2017), likely mimicking the effect of mild hypoglycaemia. Thus, Gasior and colleges concluded that the proconvulsant action might be related to restriction of glucose uptake and subsequent hypoglycaemia while anticonvulsant effects could work via the inhibition of glycolysis. Under these conditions, glucose metabolism is shunted via the pentose phosphate cycle, which has been suggested to be anticonvulsant *per se* (Lian et al., 2007). Remarkably, in this study bypassing the block of the glycolytic pathway by the administration of extrinsic lactate abolished the anticonvulsant activity of 2DG in the pilocarpine model of epilepsy. This finding further reinforces the role of intrinsic lactate as possible energy resource during seizures and not a mere byproduct, which has to be removed into the bloodstream. Alterations in the expression pattern of different MCT subtypes in chronic epileptic tissue are in favor of lactate retention (Lauritzen et al., 2012), which might explain the increased interictal lactate levels, despite of the hypometabolism (Cavus et al., 2005).

The acute anticonvulsant effects of the ketogenic diet might be also related to the fact that ketone bodies represent a bypass of glycolysis, supporting the role of lactate in seizure control (Hartman and Stafstrom, 2013). However, mimicking the ketogenic diet *in vitro* failed to prevent the occurrence of seizures (Samoilova et al., 2010). On the long term both, chronic 2DG supply and ketogenic diet seem to work by changing gene expression and the whole machinery of energy metabolism (Bough and Rho, 2007). Similar long term changes might underlie the fact that chronic metabolic support by pyruvate administration can be actually anticonvulsant in three different epilepsy models (Popova et al., 2017). As these alterations are not expected to contribute to seizure termination under normal dietary conditions, the reader is referred to excellent recent reviews on this topic.

Although the effect of MCT inhibitors was not mediated by changes in pH, the acidosis occurring during seizures is in turn capable to reduce neuronal excitability (Lux et al., 1986; Xiong et al., 2000; Wemmie et al., 2013) and by itself can exert strong anticonvulsant effects (Caspers and Speckmann, 1972). Extracellular acidosis emerges from the enhanced respiration resulting in H_2_CO_3_ formation in addition to the release of lactate (Mookerjee et al., 2015). Besides its intracellular effects (Wei et al., 2011) metabolic acidosis can alter excitability by many different ways (Sinning and Hübner, 2013), i.e., by activation of ASIC channels on interneurons (Ziemann et al., 2008) negative modulation of the *N*-methyl-D-aspartate (NMDA) receptor currents (Tang et al., 1990), inhibition of presynaptic voltage gated Ca^2+^ currents (Wemmie et al., 2013) as well as facilitation of ecto-ATPases and increasing adenosine release (Dulla et al., 2005).

Nevertheless, intracellular acidosis could directly influence GABAergic transmission as the GABA receptor channel is permeable to HCO_3_^-^ (Pavlov et al., 2013; Ruusuvuori and Kaila, 2014). Indeed, inhibitors of brain carbonic anhydrases, which actively control pH balance by catalyzing the interconversion of carbon dioxide to bicarbonate (HCO3^-^) and a proton (H^+^), possess anticonvulsant effects (Reiss and Oles, 1996; Mishra et al., 2018).

Thus, while manipulation of substrate availability and metabolic pathways could be pro and anticonvulsive, acidosis and adenosine represent two powerful and interdependent negative feedback mechanisms for intrinsic seizure termination by signalizing excessive metabolic activity before shortage of the substrates would appear (Dulla et al., 2005; Tolner et al., 2011).

## Seizure-Associated Changes in CMRO_2_ and Cerebral Blood Flow

Besides the potentially catastrophic events such as disturbances in cerebral blood flow or phenomena like spreading depolarization (Dreier, 2011), seizures represent a metabolic burden which likely pushes brain energy metabolism to its limits. This assumption is supported by the finding that energy demanding physiological-type of network activity, such as carbachol induced lasting gamma oscillations in the brain slice preparation, result in a similar increase in CMRO_2_ as observed during seizure-like activity in the same tissue (Huchzermeyer et al., 2008; Kann et al., 2011). While the enhancement of CMRO_2_ is sufficient to keep transmembrane ion-gradients constant during gamma oscillations, seizure-like events result in massive changes in ion homeostasis (Huchzermeyer et al., 2013). Thus, it is tempting to speculate that ion transport capacity -and the corresponding ATP demand- is covered by the increase in the CMRO_2_ under physiological network activity. Any further increase in ion-fluxes exceeding pump capacity would result in altered transmembrane gradients, without further induction of CMRO_2_ increase.

Seizure-associated increases in CMRO_2_ *in vivo* can be calculated from the simultaneous determination of local tissue pO_2_ and cerebral blood flow (CBF, Gjedde, 2005; Thomsen et al., 2009) or by using fMRI as CBF, CMRO_2_ and CBV are the dominant physiologic parameters that modulate the BOLD signal (Kida et al., 2000). However, these calculations has to take into account the local variability of the signals and the fact that their relation can be changed by the seizure itself. Thus under bicuculline induced lasting ictal activity a positive BOLD was observed in the cortex and a negative BOLD signal in the hippocampus, despite of the increase in CBV and neuronal activity in both regions, suggesting a local mismatch between CMRO_2_ and CBF (Schridde et al., 2008).

The brain slice preparation offers a simpler solution, because in the absence of blood flow, and permanent carbogen gassing (95%O_2_ 5%CO_2_) of the tissue, local pO_2_ depends solely on the rate of respiration and the diffusion distance from the surface (Liotta et al., 2012; Huchzermeyer et al., 2013). By recording basal and seizure-associated changes in pO_2_ and extracellular potassium/sodium concentration it was possible to calculate ATP consumption, ATP levels, CMRO_2_ and to predict the electron flow via the TCA enzymes and electron carrier nucleotides, FADH_2_ and NAD (Schoknecht et al., 2017). The transformation from interictal to seizure-like events was associated with a fivefold increase in oxygen consumption, whereas the corresponding increase in ATP demand was significantly higher, indicating that coupling ratio (ATP/O_2_) and thereby the efficiency of oxidative metabolism is improved during seizure-like events. Notably, at maximum CMRO_2_, tissue pO_2_ as well as the reducing equivalents for the ETC were not rate limiting for the energy metabolism, suggesting that shortage of these factors is not a necessary prerequisite for seizure termination (Foster et al., 2005; Ivanov et al., 2015). Although these data were obtained *in vitro*, the results on CMRO_2_ and pO_2_ distribution can be extrapolated to the *in vivo* situation by knowing the intercapillary distance and the seizure-associated changes in local cerebral blood flow response.

In general, based on the close relationship between neuronal activity and vascular response (i.e., neurovascular coupling), seizures induce reversible vasodilation and disproportionally high increases in the blood flow, resulting in an overshooting supply of oxygenated hemoglobin, underlying the seizure-associated BOLD response (Tyvaert et al., 2009; Thornton et al., 2010). The first hypotheses of functional hyperemia assumed that increased energy consumption by active neurons induce vasodilation directly by altering the concentration of extracellular potassium, oxygen, protons and different metabolites (Nilsson et al., 1978). Alternatively, neuronal activity-dependent increase of extracellular glutamate can induce the release of vasoactive substances such as NO leading to subsequent smooth muscle cell relaxation (Lauritzen, 2005; Busija et al., 2007). Astrocytes, the anatomical intermediaries between neurons and blood vessels, are important mediators of neurovascular coupling by releasing either vasodilator or vasoconstrictor agents in addition to potassium signaling at the astrocytic end-feet (Zonta et al., 2003; Filosa et al., 2006; Gordon et al., 2008; Petzold and Murthy, 2011; Filosa and Iddings, 2013). Ictal increases in parenchymal lactate concentration also contribute to the regulation of cerebral blood flow. In the presence of restricted oxygen availability and high astrocytic calcium concentrations astrocytic lactate release is maximized. The subsequent lactate accumulation attenuates transporter-mediated uptake of prostaglandin E2 (PGE2) from the extracellular space leading to subsequent vasodilation (Gordon et al., 2008). Thus, the vascular effect of lactate has to be kept in mind when considering the anticonvulsant effects of lactate uptake or glycolysis inhibitors under *in vivo* conditions. Finally, seizure-associated increases in extracellular adenosine have also vasodilatory effects by inhibiting constriction of arteriolar smooth muscle cells via adenosine receptors (Gordon et al., 2008).

The overshooting blood flow response to seizures led to the ‘unorthodox’ hypothesis that ictogenesis is actually a restorative process to increase supply in underperfused areas (Doman and Pelligra, 2003). Nevertheless, more recent results show that BOLD signal is preceded by a local pO_2_ dip and temporary reduction of the blood flow was documented in the tissue surrounding the epileptic focus as well as in the contralateral hemisphere (Zhao et al., 2011; Ma et al., 2013; Harris et al., 2014), indicating that the underperfusion *per se* is not an initiator of ictogenesis.

Status epilepticus and even a single seizure is associated with neuronal injury which in turn might favor secondary ictogenesis (Heinemann, 2004). The first clear proof of selective neuronal loss following status epilepticus appeared early in the 18th century, describing a sequence of cellular changes observed with Nissl staining in the brains of seven patients dying during the course of status epilepticus (Clark and Prout, 1903). The idea that ischemic processes could explain the cell loss in status epilepticus was developed about 100 years ago but questioned steadily based on the presence of massive hyperaemia and hyperoxygenation (Pfleger, 1880; Meldrum, 2002). Alternatively Pinard et al. (1984) suggested that not hypoxia but rather the release of endogenous substances and excitotoxic cascades mediate an excessive rise in [Ca^2+^]i resulting in cell death. Theoretically, a relative hypoperfusion of the tissue might occur also with intact neurovascular coupling if we presume that the diffusion-limited oxygen delivery can not keep up with a local increases in CMRO_2_. This kind of hypoperfusion might underlie the local negative BOLD signal indicating a mismatch between CBF and CMRO_2_ (Schridde et al., 2008). While such changes would remain undetected with the conventional doppler flowmetry (Geneslaw et al., 2011), doppler-based functional ultrasound imaging modality could reveal seizure-associated relative hypoperfusion at the microcircuitry level (Urban et al., 2017). Oxygen transport from the capillary in the tissue is driven by the oxygen gradient within the tissue. Thus an increase in CMRO_2_ would require an equal increase in the oxygen gradients. Taking into account that during high energy demand a significant part of the tissue exhibits pO_2_ below 50% compared to the capillary value (Schneider et al., 2017), the increase in capillary pO_2_ observed as a positive BOLD signal can still mean hypoxic pO_2_ at certain places within the brain parenchim. Such phenomenon was observed during spreading depolarization in the neocortex where monitoring the tissue redox potential revealed hypoperfused “islets” between capillaries (Takano et al., 2007). Similar hypoperfused areas might be present at the immediate vicinity of seizure focus, indicating altered blood distribution rather than disturbances of neurovascular coupling (Zhao et al., 2009). On the other hand, status epilepticus was shown to induce damage and to severely alter the neurovascular unit, including dysfunction of the blood-brain-barrier (BBB, Abbott and Friedman, 2012; Heinemann et al., 2012), which might also affect the functionality of neurovascular coupling and substrate supply during recurrent seizures. Thus interictal hypoperfusion is not a sole consequence of cell loss and impaired neuronal energy metabolism but it might also represent disturbances of neurovascular coupling at the microcirculation level (see next chapter).

## Uncoupling of Blood Flow and Ictal Events and Postictal Disturbances of Metabolic Supply

Neurovascular coupling necessitates information flow between neurons, astrocytes, endothelial cells and the contractile elements of the vasculature, i.e., the smooth muscle cells in arterioles and pericytes in capillaries (Petzold and Murthy, 2011; Hall et al., 2014). Disturbances in NVC has been associated with several pathologies, including stroke (Yemisci et al., 2009), hypertension (Dunn and Nelson, 2014), Alzheimer’s disease (Rancillac et al., 2012), and subarachnoid hemorrhage (Piilgaard and Lauritzen, 2009). In epilepsy, alterations in the function of the neurovascular unit were found in epileptic tissue following status epilepticus leading to BBB impairment (Gorter et al., 2015; Bar-Klein et al., 2017). Following pilocarpine-induced status epilepticus heterogeneously distributed vascular alterations were observed within the cerebral cortex (Fabene et al., 2007). Superficial arteries and veins remained intact allowing for increased blood flow, whereas microvasculature showed a reduction of the diameter, resulting in an ischemic core in the deeper layers of the cortex. Moreover, neurovascular coupling was found to be deviate from the electrical activity and even impaired following seizures (Parfenova et al., 2005, 2012; Ma et al., 2013; Harris et al., 2014), thus raising the question whether inadequate perfusion and hypoxia may contribute to the pathophysiological changes in energy metabolism following status epilepticus. Indeed, fluorescence life-time measurement of NADH revealed metabolic impairment in bicuculline induced focal seizure activity *in vivo* likely due to inadequate blood supply (Yaseen et al., 2017).

With respect to microcirculation, gap junction-coupled contractile pericytes gained importance as the key executors of neurovascular coupling at the capillaries (Peppiatt et al., 2006; Hall et al., 2014; Mishra et al., 2016). Pericytic injury has been described to be accompanied by neurovascular dysfunction in various neurological disorders(for review see Winkler et al., 2011). Pericyte degeneration induced white-matter hypoxia and loss of myelin, (Montagne et al., 2018) and peroxynitrite-dependent pericytic dysfunction could completely disrupt passage of erythrocytes in capillaries as seen in the no-reflow phenomenon following ischemia (Yemisci et al., 2009). On the other hand, Hall et al. (2014) found free radical-independent pericytic dysfunction following oxygen glucose deprivation in brain slices. Similar to hypoxia-reperfusion, epileptic seizures are also associated with increased formation of oxygen and nitrogen centered free radicals, both *in vivo* (Folbergrová et al., 2012) and *in vitro* (Kovács et al., 2002, 2009). However, it is not known whether the formation of free radicals during status epilepticus would also influence pericyte function. Nevertheless, the redistribution of pericytes have been shown after status epilepticus (Milesi et al., 2014) and pericyte-mediated capillary vasospasm was observed in a genetic model of epilepsy and in kainic acid-treated animals following seizure onset (Leal-Campanario et al., 2017). Postictal hypoxia due to cyclooxygenase-2 activity-dependent vasoconstriction has been made responsible for the debilitating consequences of seizures (Farrell et al., 2016, 2017a). Theoretically, such changes in capillary perfusion would remain undetected with conventional methods of blood flow monitoring and could contribute to the development of local under-supply in case of subsequent seizures. Altogether, these studies highlight the importance of examining the time course and development of metabolic- and neurovascular-abnormalities associated with seizures. Besides the fact that capillaries are buried deep in the brain tissue and only accessible to multiphoton imaging or endomicroscopy, the difficulty with monitoring of pericytic regulation of capillary blood flow during seizures *in vivo* is that dilations of precapillary arteries/arterioles or changes in systemic blood pressure might influence the responses of pericytes (Fernández-Klett and Priller, 2015). Alternatively, the use of *in vitro* preparation have been suggested that combine the application of haemodynamic variables (such as flow and pressure) into parenchymal arterioles with the advantages of capillary imaging in brain slices (Kim and Filosa, 2012). In order to study capillary responses and BBB function in a controlled microenvironment we established a slice culture model of organotypic microvasculature. In this experimental setting, components of the neurovascular unit and BBB remain functional (Moser et al., 2003; Camenzind et al., 2010) and pericyte-derived contractile cells regulate capillary diameter in response to vasoactive substrates and increased intramural pressure (Kovács et al., 2011). Moreover, vascular remodeling takes place upon lasting epileptiform activity, mimicking seizure-associated disturbances in neurovascular unit (Morin-Brureau et al., 2011). Investigating capillary vasomotility in this preparation could provide insights on the mechanisms of pericytic disturbances underlying neurovascular uncoupling.

## Concluding Remarks

In conclusion, substantial evidence suggests that seizure termination can occur in the presence of intact neurometabolic coupling and overshooting neurovascular responses without immediate limitation on the metabolism. On the other hand, enhanced energy metabolism exerts a strong negative feedback on neuronal excitability, mostly via adenosine and pH changes. Free radical-dependent damage of mitochondrial enzymes and the subsequent disturbances in energy metabolism observed in chronic epileptic tissue were found to be rather proconvulsive (**Figure 2**). Manipulating substrate availability, preventing glycolysis, lactate uptake as well as enhancing pentose phosphate shunt might have both acute pro- or anticonvulsant effects, whereas chronic dietary changes can exert seizure control via epigenetic mechanisms. Thus targeting negative feedback mechanisms involved in metabolism dependent regulation of excitability as well as supporting cellular bioenergetics and protecting neurovascular coupling may represent promising therapeutic approaches in the treatment of epilepsy.

## Author Contributions

RK, NB, OP, and ZG reviewed the bibliography and wrote the first draft of the manuscript. AF, SG, and JO added substantial comments after critical reading of the text.

## Conflict of Interest Statement

The authors declare that the research was conducted in the absence of any commercial or financial relationships that could be construed as a potential conflict of interest.

## References

[B1] Abbott N. J. Friedman A. (2012). Overview and introduction: the blood-brain barrier in health and disease. *Epilepsia* 53(Suppl. 6), 1–6. 10.1111/j.1528-1167.2012.03696.x 23134489 PMC3625728

[B2] Ames A. (2000). CNS energy metabolism as related to function. *Brain Res. Brain Res. Rev.* 34 42–68. 10.1016/S0165-0173(00)00038-2 11086186

[B3] Angamo E. A. Rösner J. Liotta A. Kovács R. Heinemann U. (2016). A neuronal lactate uptake inhibitor slows recovery of extracellular ion concentration changes in the hippocampal CA3 region by affecting energy metabolism. *J. Neurophysiol.* 116 2420–2430. 10.1152/jn.00327.2016 27559140 PMC5116486

[B4] Angamo E. A. ul Haq R. Rösner J. Gabriel S. Gerevich Z. Heinemann U. (2017). Contribution of intrinsic lactate to maintenance of seizure activity in neocortical slices from patients with temporal lobe epilepsy and in rat entorhinal cortex. *Int. J. Mol. Sci.* 18:1835. 10.3390/ijms18091835 28832554 PMC5618484

[B5] Avsar E. Empson R. M. (2004). Adenosine acting via A1 receptors, controls the transition to status epilepticus-like behaviour in an in vitro model of epilepsy. *Neuropharmacology* 47 427–437. 10.1016/j.neuropharm.2004.04.015 15275832

[B6] Bar-Klein G. Lublinsky S. Kamintsky L. Noyman I. Veksler R. Dalipaj H. (2017). Imaging blood-brain barrier dysfunction as a biomarker for epileptogenesis. *Brain* 140 1692–1705. 10.1093/brain/awx073 28444141

[B7] Beamer E. Otahal J. Sills G. J. Thippeswamy T. (2012). N(w) -propyl-L-arginine (L-NPA) reduces status epilepticus and early epileptogenic events in a mouse model of epilepsy: behavioural, EEG and immunohistochemical analyses. *Eur. J. Neurosci.* 36 3194–3203. 10.1111/j.1460-9568.2012.08234.x 22943535

[B8] Bedner P. Dupper A. Huttmann K. Muller J. Herde M. K. Dublin P. (2015). Astrocyte uncoupling as a cause of human temporal lobe epilepsy. *Brain* 138 1208–1222. 10.1093/brain/awv067 25765328 PMC5963418

[B9] Ben-Ari Y. (2001). Cell death and synaptic reorganizations produced by seizures. *Epilepsia* 42(Suppl. 3), 5–7. 10.1046/j.1528-1157.2001.042suppl.3005.x 11520314

[B10] Bergersen L. H. (2007). Is lactate food for neurons? Comparison of monocarboxylate transporter subtypes in brain and muscle. *Neuroscience* 145 11–19. 10.1016/j.neuroscience.2006.11.062 17218064

[B11] Bergersen L. H. Magistretti P. J. Pellerin L. (2005). Selective postsynaptic co-localization of MCT2 with AMPA receptor GluR2/3 subunits at excitatory synapses exhibiting AMPA receptor trafficking. *Cereb. Cortex* 15 361–370. 10.1093/cercor/bhh138 15749979

[B12] Berndt N. Kann O. Holzhütter H. G. (2015). Physiology-based kinetic modeling of neuronal energy metabolism unravels the molecular basis of NAD(P)H fluorescence transients. *J. Cereb. Blood Flow Metab.* 35 1494–1506. 10.1038/jcbfm.2015.70 25899300 PMC4640339

[B13] Boison D. Steinhäuser C. (2018). Epilepsy and astrocyte energy metabolism. *Glia* 66 1235–1243. 10.1002/glia.23247 29044647 PMC5903956

[B14] Bough K. J. Rho J. M. (2007). Anticonvulsant mechanisms of the ketogenic diet. *Epilepsia* 48 43–58. 10.1111/j.1528-1167.2007.00915.x 17241207

[B15] Busija D. W. Bari F. Domoki F. Louis T. (2007). Mechanisms involved in the cerebrovascular dilator effects of N-methyl-d-aspartate in cerebral cortex. *Brain Res. Rev.* 56 89–100. 10.1016/j.brainresrev.2007.05.011 17716743 PMC2174154

[B16] Calabrese V. P. Gruemer H. D. James K. Hranowsky N. DeLorenzo R. J. (1991). Cerebrospinal fluid lactate levels and prognosis in status epilepticus. *Epilepsia* 32 816–821. 10.1111/j.1528-1157.1991.tb05538.x 1743153

[B17] Camenzind R. S. Chip S. Gutmann H. (2010). Preservation of transendothelial glucose transporter 1 and P-glycoprotein transporters in a cortical slice culture model of the blood-brain barrier. *Neuroscience* 170 361–371. 10.1016/j.neuroscience.2010.06.073 20603190

[B18] Caspers H. Speckmann E. J. (1972). Cerebral pO2, pCO2 and pH: changes during convulsive activity and their significance for spontaneous arrest of seizures. *Epilepsia* 13 699–725. 10.1111/j.1528-1157.1972.tb04403.x 4343685

[B19] Cavus I. Kasoff W. S. Cassaday M. P. Jacob R. Gueorguieva R. Sherwin R. S. (2005). Extracellular metabolites in the cortex and hippocampus of epileptic patients. *Ann. Neurol.* 57 226–235. 10.1002/ana.20380 15668975

[B20] Cendes F. Caramanos Z. Andermann F. Dubeau F. Arnold D. L. (1997). Proton magnetic resonance spectroscopic imaging and magnetic resonance imaging volumetry in the lateralization of temporal lobe epilepsy: a series of 100 patients. *Ann. Neurol.* 42 737–746. 10.1002/ana.410420510 9392573

[B21] Clark L. P. Prout T. P. (1903). Status epilepticus: a clinical and pathological study in epilepsy. *Am. J. Psychiatry* 60 645–698. 10.1176/ajp.61.1.81

[B22] Cunha R. A. (2001). Adenosine as a neuromodulator and as a homeostatic regulator in the nervous system: different roles, different sources and different receptors. *Neurochem. Int.* 38 107–125. 10.1016/S0197-0186(00)00034-6 11137880

[B23] Denton R. M. (2009). Regulation of mitochondrial dehydrogenases by calcium ions. *Biochim. Biophys. Acta* 1787 1309–1316. 10.1016/j.bbabio.2009.01.005 19413950

[B24] Dias R. B. Rombo D. M. Ribeiro J. A. Henley J. M. Sebastião A. M. (2013). Adenosine: setting the stage for plasticity. *Trends Neurosci.* 36 248–257. 10.1016/j.tins.2012.12.003 23332692

[B25] Dienel G. A. (2017). Lack of appropriate stoichiometry: strong evidence against an energetically important astrocyte-neuron lactate shuttle in brain. *J. Neurosci. Res.* 95 2103–2125. 10.1002/jnr.24015 28151548

[B26] Doğan E. A. Ünal A. Ünal A. Erdoğan Ç. (2017). Clinical utility of serum lactate levels for differential diagnosis of generalized tonic-clonic seizures from psychogenic nonepileptic seizures and syncope. *Epilepsy Behav.* 75 13–17. 10.1016/j.yebeh.2017.07.003 28806632

[B27] Doman G. Pelligra R. (2003). Ictogenesis: the origin of seizures in humans. A new look at an old theory. *Med. Hypotheses* 60 129–132. 10.1016/S0306-9877(02)00348-1 12450780

[B28] Dreier J. P. (2011). The role of spreading depression, spreading depolarization and spreading ischemia in neurological disease. *Nat. Med.* 17 439–447. 10.1038/nm.2333 21475241

[B29] Dulla C. G. Dobelis P. Pearson T. Frenguelli B. G. Staley K. J. Masino S. A. (2005). Adenosine and ATP link PCO2 to cortical excitability via pH. *Neuron* 48 1011–1023. 10.1016/j.neuron.2005.11.009 16364904 PMC1924599

[B30] Dunn K. M. Nelson M. T. (2014). Neurovascular signaling in the brain and the pathological consequences of hypertension. *Am. J. Physiol. Heart Circ. Physiol.* 306 H1–H14. 10.1152/ajpheart.00364.2013 24163077 PMC3920149

[B31] Dunwiddie T. V. (1980). Endogenously released adenosine regulates excitability in the in vitro hippocampus. *Epilepsia* 21 541–548. 10.1111/j.1528-1157.1980.tb04305.x 7418669

[B32] During M. J. Spencer D. D. (1992). Adenosine: a potential mediator of seizure arrest and postictal refractoriness. *Ann. Neurol.* 32 618–624. 10.1002/ana.410320504 1449242

[B33] Essig C. F. Flanary H. G. (1966). The importance of the convulsion in occurrence and rate of development of electroconvulsive threshold elevation. *Exp. Neurol.* 14 448–452. 10.1016/0014-4886(66)90129-4 4378199

[B34] Fabene P. F. Merigo F. Galiè M. Benati D. Bernardi P. Farace P. (2007). Pilocarpine-induced status epilepticus in rats involves ischemic and excitotoxic mechanisms. *PLoS One* 2:e1105. 10.1371/journal.pone.0001105 17971868 PMC2040510

[B35] Farrell J. S. Colangeli R. Wolff M. D. Wall A. K. Phillips T. J. George A. (2017a). Postictal hypoperfusion/hypoxia provides the foundation for a unified theory of seizure-induced brain abnormalities and behavioral dysfunction. *Epilepsia* 58 1493–1501. 10.1111/epi.13827 28632329

[B36] Farrell J. S. Gaxiola-Valdez I. Wolff M. D. David L. S. Dika H. I. Geeraert B. L. (2016). Postictal behavioural impairments are due to a severe prolonged hypoperfusion/hypoxia event that is COX-2 dependent. *eLife* 5:e19352. 10.7554/eLife.19352 27874832 PMC5154758

[B37] Farrell J. S. Wolff M. D. Teskey G. C. (2017b). Neurodegeneration and pathology in epilepsy: clinical and basic perspectives. *Adv. Neurobiol.* 15 317–334. 10.1007/978-3-319-57193-5_12 28674987

[B38] Fedele D. E. Li T. Lan J. Q. Fredholm B. B. Boison D. (2006). Adenosine A1 receptors are crucial in keeping an epileptic focus localized. *Exp. Neurol.* 200 184–190. 10.1016/j.expneurol.2006.02.133 16750195

[B39] Fernández-Klett F. Priller J. (2015). Diverse functions of pericytes in cerebral blood flow regulation and ischemia. *J. Cereb. Blood Flow Metab.* 35 883–887. 10.1038/jcbfm.2015.60 25853910 PMC4640260

[B40] Filosa J. A. Bonev A. D. Straub S. V. Meredith A. L. Wilkerson M. K. Aldrich R. W. (2006). Local potassium signaling couples neuronal activity to vasodilation in the brain. *Nat. Neurosci.* 9 1397–1403. 10.1038/nn1779 17013381

[B41] Filosa J. A. Iddings J. A. (2013). Astrocyte regulation of cerebral vascular tone. *Am. J. Physiol. Heart Circ. Physiol.* 305 H609–H619. 10.1152/ajpheart.00359.2013 23792684 PMC3761330

[B42] Fisher R. S. Acevedo C. Arzimanoglou A. Bogacz A. Cross J. H. Elger C. E. (2014). ILAE official report: a practical clinical definition of epilepsy. *Epilepsia* 55 475–482. 10.1111/epi.12550 24730690

[B43] Fisher R. S. van Emde Boas W. Blume W. Elger C. Genton P. Lee P. (2005). Epileptic seizures and epilepsy: definitions proposed by the international league against epilepsy (ILAE) and the International Bureau for Epilepsy (IBE). *Epilepsia* 46 470–472. 10.1111/j.0013-9580.2005.66104.x 15816939

[B44] Folbergrová J. Haugvicová R. Mares P. (2000). Behavioral and metabolic changes in immature rats during seizures induced by homocysteic acid: the protective effect of NMDA and non-NMDA receptor antagonists. *Exp. Neurol.* 161 336–345. 10.1006/exnr.1999.7264 10683299

[B45] Folbergrová J. Kunz W. S. (2012). Mitochondrial dysfunction in epilepsy. *Mitochondrion* 12 35–40. 10.1016/j.mito.2011.04.004 21530687

[B46] Folbergrová J. Otáhal J. Druga R. (2012). Brain superoxide anion formation in immature rats during seizures: protection by selected compounds. *Exp. Neurol.* 233 421–429. 10.1016/j.expneurol.2011.11.009 22108622

[B47] Foster K. A. Beaver C. J. Turner D. A. (2005). Interaction between tissue oxygen tension and NADH imaging during synaptic stimulation and hypoxia in rat hippocampal slices. *Neuroscience* 132 645–657. 10.1016/j.neuroscience.2005.01.040 15837126

[B48] Frantseva M. V. Velazquez J. L. Hwang P. A. Carlen P. L. (2000). Free radical production correlates with cell death in an in vitro model of epilepsy. *Eur. J. Neurosci.* 12 1431–1439. 10.1046/j.1460-9568.2000.00016.x 10762371

[B49] Galeffi F. Foster K. A. Sadgrove M. P. Beaver C. J. Turner D. A. (2007). Lactate uptake contributes to the NAD(P)H biphasic response and tissue oxygen response during synaptic stimulation in area CA1 of rat hippocampal slices. *J. Neurochem.* 103 2449–2461. 10.1111/j.1471-4159.2007.04939.x 17931363 PMC3340603

[B50] Galow L. V. Schneider J. Lewen A. Ta T. T. Papageorgiou I. E. Kann O. (2014). Energy substrates that fuel fast neuronal network oscillations. *Front. Neurosci.* 5:398. 10.3389/fnins.2014.00398 25538552 PMC4256998

[B51] Gasior M. Yankura J. Hartman A. L. French A. Rogawski M. A. (2010). Anticonvulsant and proconvulsant actions of 2-deoxy-D-glucose. *Epilepsia* 51 1385–1394. 10.1111/j.1528-1167.2010.02593.x 20491877

[B52] Gellerich F. N. Gizatullina Z. Trumbekaite S. Korzeniewski B. Gaynutdinov T. Seppet E. (2012). Cytosolic Ca2 + regulates the energization of isolated brain mitochondria by formation of pyruvate through the malate-aspartate shuttle. *Biochem. J.* 443 747–755. 10.1042/BJ20110765 22295911

[B53] Geneslaw A. S. Zhao M. Ma H. Schwartz T. H. (2011). Tissue hypoxia correlates with intensity of interictal spikes. *J. Cereb. Blood Flow Metab.* 31 1394–1402. 10.1038/jcbfm.2011.16 21343943 PMC3130319

[B54] Gjedde A. (2005). “The pathways of oxygen in brain I,” in *Oxygen Transport to Tissue XXVI. Advances in Experimental Medicine and Biology* Vol. 566 eds P. Okunieff J. Williams Y. Chen (Boston, MA: Springer).

[B55] Gordon G. R. Choi H. B. Rungta R. L. Ellis-Davies G. C. MacVicar B. A. (2008). Brain metabolism dictates the polarity of astrocyte control over arterioles. *Nature* 456 745–749. 10.1038/nature07525 18971930 PMC4097022

[B56] Gorter J. A. van Vliet E. A. Aronica E. (2015). Status epilepticus, blood-brain barrier disruption, inflammation, and epileptogenesis. *Epilepsy Behav.* 49 13–16. 10.1016/j.yebeh.2015.04.047 25958228

[B57] Guillon B. Duncan R. Biraben A. Bernard A. M. Vignal J. P. Chauvel P. (1998). Correlation between interictal regional cerebral blood flow and depth-recorded interictal spiking in temporal lobe epilepsy. *Epilepsia* 39 67–76. 10.1111/j.1528-1157.1998.tb01276.x 9578015

[B58] Haas H. L. Greene R. W. (1984). Adenosine enhances afterhyperpolarization and accommodation in hippocampal pyramidal cells. *Pflugers Arch.* 402 244–247. 10.1007/BF00585506 6097865

[B59] Hall C. N. Klein-Flügge M. C. Howarth C. Attwell D. (2012). Oxidative phosphorylation, not glycolysis, powers presynaptic and postsynaptic mechanisms underlying brain information processing. *J. Neurosci.* 32 8940–8951. 10.1523/JNEUROSCI.0026-12.2012 22745494 PMC3390246

[B60] Hall C. N. Reynell C. Gesslein B. Hamilton N. B. Mishra A. Sutherland B. A. (2014). Capillary pericytes regulate cerebral blood flow in health and disease. *Nature* 508 55–60. 10.1038/nature13165 24670647 PMC3976267

[B61] Hamil N. E. Cock H. R. Walker M. C. (2012). Acute down-regulation of adenosine A(1) receptor activity in status epilepticus. *Epilepsia* 53 177–188. 10.1111/j.1528-1167.2011.03340.x 22150479

[B62] Harris S. Boorman L. Bruyns-Haylett M. Kennerley A. Ma H. Zhao M. (2014). Contralateral dissociation between neural activity and cerebral blood volume during recurrent acute focal neocortical seizures. *Epilepsia* 55 1423–1430. 10.1111/epi.12726 25053117 PMC4336552

[B63] Hartman A. L. Stafstrom C. E. (2013). Harnessing the power of metabolism for seizure prevention: focus on dietary treatments. *Epilepsy Behav.* 26 266–272. 10.1016/j.yebeh.2012.09.019 23110824 PMC3562425

[B64] Heinemann U. (2004). Basic mechanisms of partial epilepsies. *Curr. Opin. Neurol.* 17 155–159. 10.1097/00019052-200404000-00012 15021242

[B65] Heinemann U. Buchheim K. Gabriel S. Kann O. Kovács R. Schuchmann S. (2002). Coupling of electrical and metabolic activity during epileptiform discharges. *Epilepsia* 43(Suppl. 5), 168–173. 10.1046/j.1528-1157.43.s.5.15.x 12121315

[B66] Heinemann U. Kaufer D. Friedman A. (2012). ). Blood-brain barrier dysfunction, TGFβ signaling, and astrocyte dysfunction in epilepsy. *Glia* 60 1251–1257. 10.1002/glia.22311 22378298 PMC3615248

[B67] Hetherington H. Kuzniecky R. Pan J. Mason G. Morawetz R. Harris C. (1995). Proton nuclear magnetic resonance spectroscopic imaging of human temporal lobe epilepsy at 4.1 T. *Ann. Neurol.* 38 396–404. 10.1002/ana.410380309 7668825

[B68] Hildebrand M. S. Damiano J. A. Mullen S. A. Bellows S. T. Oliver K. L. Dahl H. H. (2014). Glucose metabolism transporters and epilepsy: only GLUT1 has an established role. *Epilepsia* 55 e18–e21. 10.1111/epi.12519 24483274

[B69] Hong S. B. Han H. J. Roh S. Y. Seo D. W. Kim S. E. Kim M. H. (2002). Hypometabolism and interictal spikes during positron emission tomography scanning in temporal lobe epilepsy. *Eur. Neurol.* 48 65–70. 10.1159/000062985 12186995

[B70] Hu H. Roth F. C. Vandael D. Jonas P. (2018). Complementary tuning of Na + and K + channel gating underlies fast and energy-efficient action potentials in GABAergic interneuron axons. *Neuron* 98 156.e5–165.e6. 10.1016/j.neuron.2018.02.024 29621485 PMC5896255

[B71] Huchzermeyer C. Albus K. Gabriel H. J. Otáhal J. Taubenberger N. Heinemann U. (2008). Gamma oscillations and spontaneous network activity in the hippocampus are highly sensitive to decreases in pO2 and concomitant changes in mitochondrial redox state. *J. Neurosci.* 28 1153–1162. 10.1523/JNEUROSCI.4105-07.2008 18234893 PMC6671409

[B72] Huchzermeyer C. Berndt N. Holzhütter H. G. Kann O. (2013). Oxygen consumption rates during three different neuronal activity states in the hippocampal CA3 network. *J. Cereb. Blood Flow Metab.* 33 263–271. 10.1038/jcbfm.2012.165 23168532 PMC3564197

[B73] Ingvar M. (1986). Cerebral blood flow and metabolic rate during seizures. Relationship to epileptic brain damage. *Ann. N. Y. Acad. Sci.* 462 194–206. 10.1111/j.1749-6632.1986.tb51254.x 3518570

[B74] Ivanov A. I. Bernard C. Turner D. A. (2015). Metabolic responses differentiate between interictal, ictal and persistent epileptiform activity in intact, immature hippocampus in vitro. *Neurobiol. Dis.* 75 1–14. 10.1016/j.nbd.2014.12.013 25533681 PMC4351116

[B75] Kang H. C. Lee Y. M. Kim H. D. (2013). Mitochondrial disease and epilepsy. *Brain Dev.* 35 757–761. 10.1016/j.braindev.2013.01.006 23414619

[B76] Kann O. Huchzermeyer C. Kovács R. Wirtz S. Schuelke M. (2011). Gamma oscillations in the hippocampus require high complex I gene expression and strong functional performance of mitochondria. *Brain* 134(Pt 2), 345–358. 10.1093/brain/awq333 21183487

[B77] Kann O. Kovács R. Njunting M. Behrens C. J. Otáhal J. Lehmann T. N. (2005). Metabolic dysfunction during neuronal activation in the ex vivo hippocampus from chronic epileptic rats and humans. *Brain* 128(Pt 10), 2396–2407. 10.1093/brain/awh568 15958506

[B78] Kannurpatti S. S. (2017). Mitochondrial calcium homeostasis: implications for neurovascular and neurometabolic coupling. *J. Cereb. Blood Flow Metab.* 37 381–395. 10.1177/0271678X16680637 27879386 PMC5381466

[B79] Kasischke K. A. Vishwasrao H. D. Fisher P. J. Zipfel W. R. Webb W. W. (2004). Neural activity triggers neuronal oxidative metabolism followed by astrocytic glycolysis. *Science* 2 99–103. 10.1126/science.1096485 15232110

[B80] Kida I. Kennan R. P. Rothman D. L. Behar K. L. Hyder F. (2000). High-resolution CMR(O2) mapping in rat cortex: a multiparametric approach to calibration of BOLD image contrast at 7 Tesla. *J. Cereb. Blood Flow Metab.* 20 847–860. 10.1097/00004647-200005000-00012 10826536

[B81] Kim K. J. Filosa J. A. (2012). Advanced in vitro approach to study neurovascular coupling mechanisms in the brain microcirculation. *J. Physiol.* 590 1757–1770. 10.1113/jphysiol.2011.222778 22310311 PMC3413490

[B82] Kirchner A. Velísková J. Velísek L. (2006). Differential effects of low glucose concentrations on seizures and epileptiform activity in vivo and in vitro. *Eur. J. Neurosci.* 23 1512–1522. 10.1111/j.1460-9568.2006.04665.x 16553614

[B83] Klaft Z. J. Hollnagel J. O. Salar S. Calişkan G. Schulz S. B. Schneider U. C. (2016). Adenosine A1 receptor-mediated suppression of carbamazepine-resistant seizure-like events in human neocortical slices. *Epilepsia* 57 746–756. 10.1111/epi.13360 27087530

[B84] Klaft Z. J. Schulz S. B. Maslarova A. Gabriel S. Heinemann U. Gerevich Z. (2012). Extracellular ATP differentially affects epileptiform activity via purinergic P2X7 and adenosine A1 receptors in naive and chronic epileptic rats. *Epilepsia* 53 1978–1986. 10.1111/j.1528-1167.2012.03724.x 23106524

[B85] Kochanek P. M. Vagni V. A. Janesko K. L. Washington C. B. Crumrine P. K. Garman R. H. (2006). Adenosine A1 receptor knockout mice develop lethal status epilepticus after experimental traumatic brain injury. *J. Cereb. Blood Flow Metab.* 26 565–575. 10.1038/sj.jcbfm.9600218 16121125

[B86] Kovac S. Domijan A. M. Walker M. C. Abramov A. Y. (2012). Prolonged seizure activity impairs mitochondrial bioenergetics and induces cell death. *J. Cell Sci.* 125(Pt 7), 1796–1806. 10.1242/jcs.099176 22328526 PMC4195235

[B87] Kovács R. Heinemann U. (2014). Models in research of pharmacoresistant epilepsy: present and future in development of antiepileptic drugs. *Curr. Med. Chem.* 21 689–703. 10.2174/0929867320666131119152613 24251565

[B88] Kovács R. Kardos J. Heinemann U. Kann O. (2005). Mitochondrial calcium ion and membrane potential transients follow the pattern of epileptiform discharges in hippocampal slice cultures. *J. Neurosci.* 25 4260–4269. 10.1523/JNEUROSCI.4000-04.2005 15858052 PMC6725115

[B89] Kovács R. Papageorgiou I. Heinemann U. (2011). Slice cultures as a model to study neurovascular coupling and blood brain barrier in vitro. *Cardiovasc. Psychiatry Neurol.* 2011:646958. 10.1155/2011/646958 21350722 PMC3042620

[B90] Kovács R. Rabanus A. Otáhal J. Patzak A. Kardos J. Albus K. (2009). Endogenous nitric oxide is a key promoting factor for initiation of seizure-like events in hippocampal and entorhinal cortex slices. *J. Neurosci.* 29 8565–8577. 10.1523/JNEUROSCI.5698-08.2009 19571147 PMC6665664

[B91] Kovács R. Schuchmann S. Gabriel S. Kann O. Kardos J. Heinemann U. (2002). Free radical-mediated cell damage after experimental status epilepticus in hippocampal slice cultures. *J. Neurophysiol.* 88 2909–2918. 10.1152/jn.00149.2002 12466417

[B92] Kovács R. Schuchmann S. Gabriel S. Kardos J. Heinemann U. (2001). Ca2 + signalling and changes of mitochondrial function during low-Mg2 + -induced epileptiform activity in organotypic hippocampal slice cultures. *Eur. J. Neurosci.* 13 1311–1319. 10.1046/j.0953-816x.2001.01505.x 11298791

[B93] Kunz W. S. Kudin A. P. Vielhaber S. Blümcke I. Zuschratter W. Schramm J. (2000). Mitochondrial complex I deficiency in the epileptic focus of patients with temporal lobe epilepsy. *Ann. Neurol.* 48 766–773. 10.1002/1531-8249(200011)48:5<766::AID-ANA10>3.0.CO;2-M 11079540

[B94] Kuroda Y. (1978). Physiological roles of adenosine derivatives which are released during neurotransmission in mammalian brain. *J. Physiol.* 74 463–470. 217994

[B95] Lauritzen F. Heuser K. de Lanerolle N. C. Lee T. S. Spencer D. D. Kim J. H. (2012). Redistribution of monocarboxylate transporter 2 on the surface of astrocytes in the human epileptogenic hippocampus. *Glia* 60 1172–1181. 10.1002/glia.22344 22535546 PMC3664041

[B96] Lauritzen M. (2005). Reading vascular changes in brain imaging: is dendritic calcium the key? *Nat. Rev. Neurosci.* 6 77–85. 10.1038/nrn1589 15611729

[B97] Leal-Campanario R. Alarcon-Martinez L. Rieiro H. Martinez-Conde S. Alarcon-Martinez T. Zhao X. (2017). Abnormal capillary vasodynamics contribute to ictal neurodegeneration in epilepsy. *Sci. Rep.* 2017:43276. 10.1038/srep43276 28240297 PMC5327474

[B98] Ledo A. Barbosa R. Cadenas E. Laranjinha J. (2010). Dynamic and interacting profiles of ^∗^NO and O2 in rat hippocampal slices. *Free Radic. Biol. Med.* 48 1044–1050. 10.1016/j.freeradbiomed.2010.01.024 20100565 PMC2839026

[B99] Lee Y. Morrison B. M. Li Y. Lengacher S. Farah M. H. Hoffman P. N. (2012). Oligodendroglia metabolically support axons and contribute to neurodegeneration. *Nature* 487 443–448. 10.1038/nature11314 22801498 PMC3408792

[B100] Lian X. Y. Khan F. A. Stringer J. L. (2007). Fructose-1,6-bisphosphate has anticonvulsant activity in models of acute seizures in adult rats. *J. Neurosci.* 27 12007–12011. 10.1523/JNEUROSCI.3163-07.2007 17978042 PMC6673383

[B101] Liotta A. Rösner J. Huchzermeyer C. Wojtowicz A. Kann O. Schmitz D. (2012). Energy demand of synaptic transmission at the hippocampal Schaffer-collateral synapse. *J. Cereb. Blood Flow Metab.* 32 2076–2083. 10.1038/jcbfm.2012.116 22929439 PMC3493999

[B102] Löscher W. Köhling R. (2010). Functional, metabolic, and synaptic changes after seizures as potential targets for antiepileptic therapy. *Epilepsy Behav.* 19 105–113. 10.1016/j.yebeh.2010.06.035 20705520

[B103] Lovatt D. Xu Q. Liu W. Takano T. Smith N. A. Schnermann J. (2012). Neuronal adenosine release, and not astrocytic ATP release, mediates feedback inhibition of excitatory activity. *Proc. Natl. Acad. Sci. U.S.A.* 109 6265–6270. 10.1073/pnas.1120997109 22421436 PMC3341061

[B104] Lux H. D. Heinemann U. Dietzel I. (1986). Ionic changes and alterations in the size of the extracellular space during epileptic activity. *Adv. Neurol.* 44 619–639. 3518349

[B105] Ma H. Zhao M. Schwartz T. H. (2013). Dynamic neurovascular coupling and uncoupling during ictal onset, propagation, and termination revealed by simultaneous in vivo optical imaging of neural activity and local blood volume. *Cereb. Cortex* 23 885–899. 10.1093/cercor/bhs079 22499798 PMC3593576

[B106] Malinska D. Kulawiak B. Kudin A. P. Kovacs R. Huchzermeyer C. Kann O. (2010). Complex III-dependent superoxide production of brain mitochondria contributes to seizure-related ROS formation. *Biochim. Biophys. Acta* 1797 1163–1170. 10.1016/j.bbabio.2010.03.001 20211146

[B107] McCormack J. G. Halestrap A. P. Denton R. M. (1990). Role of calcium ions in regulation of mammalian intramitochondrial metabolism. *Physiol. Rev.* 70 391–425. 10.1152/physrev.1990.70.2.391 2157230

[B108] Meldrum B. S. (2002). Concept of activity-induced cell death in epilepsy: historical and contemporary perspectives. *Prog. Brain Res.* 135 3–11. 10.1016/S0079-6123(02)35003-9 12143350

[B109] Meric P. Barrere B. Peres M. Gillet B. Berenger G. Beloeil J. C. (1994). Effects of kainate-induced seizures on cerebral metabolism: a combined 1H and 31P NMR study in rat. *Brain Res.* 638 53–60. 10.1016/0006-8993(94)90632-7 8199876

[B110] Milesi S. Boussadia B. Plaud C. Catteau M. Rousset M. C. De Bock F. (2014). Redistribution of PDGFRβ cells and NG2DsRed pericytes at the cerebrovasculature after status epilepticus. *Neurobiol. Dis.* 71 151–158. 10.1016/j.nbd.2014.07.010 25088711 PMC4179992

[B111] Mishra A. Reynolds J. P. Chen Y. Gourine A. V. Rusakov D. A. Attwell D. (2016). Astrocytes mediate neurovascular signaling to capillary pericytes but not to arterioles. *Nat. Neurosci.* 19 1619–1627. 10.1038/nn.4428 27775719 PMC5131849

[B112] Mishra C. B. Kumari S. Angeli A. Bua S. Tiwari M. Supuran C. T. (2018). Discovery of benzenesulfonamide derivatives as carbonic anhydrase inhibitors with effective anticonvulsant action: design, synthesis, and pharmacological evaluation. *J. Med. Chem.* 61 3151–3165. 10.1021/acs.jmedchem.8b00208 29566486

[B113] Mogul D. J. Adams M. E. Fox A. P. (1993). Differential activation of adenosine receptors decreases N-type but potentiates P-type Ca2 + current in hippocampal CA3 neurons. *Neuron* 10 327–334. 10.1016/0896-6273(93)90322-I 8382501

[B114] Montagne A. Nikolakopoulou A. M. Zhao Z. Sagare A. P. Si G. Lazic D. (2018). Pericyte degeneration causes white matter dysfunction in the mouse central nervous system. *Nat. Med.* 24 326–337. 10.1038/nm.4482 29400711 PMC5840035

[B115] Mookerjee S. A. Goncalves R. L. S. Gerencser A. A. Nicholls D. G. Brand M. D. (2015). The contributions of respiration and glycolysis to extracellular acid production. *Biochim. Biophys. Acta* 1847 171–181. 10.1016/j.bbabio.2014.10.005 25449966

[B116] Morin-Brureau M. Lebrun A. Rousset M. C. Fagni L. Bockaert J. de Bock F. (2011). Epileptiform activity induces vascular remodeling and zonula occludens 1 downregulation in organotypic hippocampal cultures: role of VEGF signaling pathways. *J. Neurosci.* 31 10677–10688. 10.1523/JNEUROSCI.5692-10.2011 21775611 PMC6622643

[B117] Moser K. V. Schmidt-Kastner R. Hinterhuber H. Humpel C. (2003). Brain capillaries and cholinergic neurons persist in organotypic brain slices in the absence of blood flow. *Eur. J. Neurosci.* 18 85–94. 10.1046/j.1460-9568.2003.02728.x 12859340

[B118] Nagase M. Takahashi Y. Watabe A. M. Kubo Y. Kato F. (2014). On-site energy supply at synapses through monocarboxylate transporters maintains excitatory synaptic transmission. *J. Neurosci.* 34 2605–2617. 10.1523/JNEUROSCI.4687-12.2014 24523550 PMC6802746

[B119] Nicholls D. G. (2017). Brain mitochondrial calcium transport: origins of the set-point concept and its application to physiology and pathology. *Neurochem. Int.* 109 5–12. 10.1016/j.neuint.2016.12.018 28057556

[B120] Nilsson B. Rehncrona S. Siesjö B. K. (1978). Coupling of cerebral metabolism and blood flow in epileptic seizures, hypoxia and hypoglycaemia. *Ciba Found. Symp.* 56 199–218. 10.1002/9780470720370.ch11 27337

[B121] Otáhal J. Folbergrová J. Kovacs R. Kunz W. S. Maggio N. (2014). Epileptic focus and alteration of metabolism. *Int. Rev. Neurobiol.* 114 209–243. 10.1016/B978-0-12-418693-4.00009-1 25078504

[B122] Parfenova H. Carratu P. Tcheranova D. Fedinec A. Pourcyrous M. Leffler C. W. (2005). Epileptic seizures cause extended postictal cerebral vascular dysfunction that is prevented by HO-1 overexpression. *Am. J. Physiol. Heart Circ. Physiol.* 288 H2843–H2850. 10.1152/ajpheart.01274.2004 15681702

[B123] Parfenova H. Leffler C. W. Basuroy S. Liu J. Fedinec A. L. (2012). Antioxidant roles of heme oxygenase, carbon monoxide, and bilirubin in cerebral circulation during seizures. *J. Cereb. Blood Flow Metab.* 32 1024–1034. 10.1038/jcbfm.2012.13 22354150 PMC3367218

[B124] Pavlov I. Kaila K. Kullmann D. M. Miles R. (2013). Cortical inhibition, pH and cell excitability in epilepsy: what are optimal targets for antiepileptic interventions? *J. Physiol.* 591 765–774. 10.1113/jphysiol.2012.237958 22890709 PMC3591695

[B125] Pellerin L. Magistretti P. J. (2012). Sweet sixteen for ANLS. *J. Cereb. Blood Flow Metab.* 32 1152–1166. 10.1038/jcbfm.2011 22027938 PMC3390819

[B126] Peppiatt C. M. Howarth C. Mobbs P. Attwell D. (2006). Bidirectional control of CNS capillary diameter by pericytes. *Nature* 443 700–704. 10.1038/nature05193 17036005 PMC1761848

[B127] Petzold G. C. Murthy V. N. (2011). Role of astrocytes in neurovascular coupling. *Neuron* 71 782–797. 10.1016/j.neuron.2011.08.009 21903073

[B128] Pfleger L. (1880). Beobachtungen uber schrumpfung und skierose des ammonshorns bei epilepsie. *Allg. Z. Psychiatr.* 36 359–365.

[B129] Pierre K. Pellerin L. (2005). Monocarboxylate transporters in the central nervous system: distribution, regulation and function. *J. Neurochem.* 94 1–14. 10.1111/j.1471-4159.2005.03168.x 15953344

[B130] Pietersen A. N. Lancaster D. M. Patel N. Hamilton J. B. Vreugdenhil M. (2009). Modulation of gamma oscillations by endogenous adenosine through A1 and A2A receptors in the mouse hippocampus. *Neuropharmacology* 56 481–492. 10.1016/j.neuropharm.2008.10.001 18955071

[B131] Piilgaard H. Lauritzen M. (2009). Persistent increase in oxygen consumption and impaired neurovascular coupling after spreading depression in rat neocortex. *J. Cereb. Blood Flow Metab.* 29 1517–1527. 10.1038/jcbfm.2009.73 19513087

[B132] Pinard E. Tremblay E. Ben-Ari Y. Seylaz J. (1984). Blood flow compensates oxygen demand in the vulnerable CA3 region of the hippocampus during kainate-induced seizures. *Neuroscience* 13 1039–1049. 10.1016/0306-4522(84)90287-2 6441898

[B133] Popova I. Malkov A. Ivanov A. I. Samokhina E. Buldakova S. Gubkina O. (2017). Metabolic correction by pyruvate halts acquired epilepsy in multiple rodent models. *Neurobiol. Dis.* 106 244–254. 10.1016/j.nbd.2017.07.012 28709994

[B134] Pumain R. Ahmed M. S. Kurcewicz I. Trottier S. Louvel J. Turak B. (2008). Lability of GABAA receptor function in human partial epilepsy: possible relationship to hypometabolism. *Epilepsia* 49(Suppl. 8), 87–90. 10.1111/j.1528-1167.2008.01845.x 19049598

[B135] Rancillac A. Geoffroy H. Rossier J. (2012). Impaired neurovascular coupling in the APPxPS1 mouse model of Alzheimer’s disease. *Curr. Alzheimer Res.* 9 1221–1230. 10.2174/156720512804142859 22799606

[B136] Reiss W. G. Oles K. S. (1996). Acetazolamide in the treatment of seizures. *Ann. Pharmacother.* 30 514–519. 10.1177/106002809603000515 8740334

[B137] Rouach N. Koulakoff A. Abudara V. Willecke K. Giaume C. (2008). Astroglial metabolic networks sustain hippocampal synaptic transmission. *Science* 322 1551–1555. 10.1126/science.1164022 19056987

[B138] Rowley S. Patel M. (2013). Mitochondrial involvement and oxidative stress in temporal lobe epilepsy. *Free Radic. Biol. Med.* 62 121–131. 10.1016/j.freeradbiomed.2013.02.002 23411150 PMC4043127

[B139] Rueda C. B. Llorente-Folch I. Amigo I. Contreras L. González-Sánchez P. Martínez-Valero P. (2014). Ca(2 + ) regulation of mitochondrial function in neurons. *Biochim. Biophys. Acta* 1837 1617–1624. 10.1016/j.bbabio.2014.04.010 24820519

[B140] Ruusuvuori E. Kaila K. (2014). Carbonic anhydrases and brain pH in the control of neuronal excitability. *Subcell Biochem.* 75 271–290. 10.1007/978-94-007-7359-2_14 24146384

[B141] Sada N. Lee S. Katsu T. Otsuki T. Inoue T. (2015). Epilepsy treatment. Targeting LDH enzymes with a stiripentol analog to treat epilepsy. *Science* 347 1362–1367. 10.1126/science.aaa1299 25792327

[B142] Samoilova M. Weisspapir M. Abdelmalik P. Velumian A. A. Carlen P. L. (2010). Chronic in vitro ketosis is neuroprotective but not anti-convulsant. *J. Neurochem.* 113 826–835. 10.1111/j.1471-4159.2010.06645.x 20163521

[B143] Samokhina E. Popova I. Malkov A. Ivanov A. I. Papadia D. Osypov A. (2017). Chronic inhibition of brain glycolysis initiates epileptogenesis. *J. Neurosci. Res.* 95 2195–2206. 10.1002/jnr.24019 28150440

[B144] Schneider J. Berndt N. Papageorgiou I. E. Maurer J. Bulik S. Both M. (2017). Local oxygen homeostasis during various neuronal network activity states in the mouse hippocampus. *J. Cereb. Blood Flow Metab.* 10.1177/0271678X17740091 [Epub ahead of print]. 29099662 PMC6501513

[B145] Schoknecht K. Berndt N. Rösner J. Heinemann U. Dreier J. P. Kovács R. (2017). Event-Associated oxygen consumption rate increases ca. five-fold when interictal activity transforms into seizure-like events In Vitro. *Int. J. Mol. Sci.* 18:E1925. 10.3390/ijms18091925 28880249 PMC5618574

[B146] Schridde U. Khubchandani M. Motelow J. E. Sanganahalli B. G. Hyder F. Blumenfeld H. (2008). Negative BOLD with large increases in neuronal activity. *Cereb. Cortex* 18 1814–1827. 10.1093/cercor/bhm208 18063563 PMC2790390

[B147] Schuchmann S. Albrecht D. Heinemann U. von Bohlen Halbach O. (2002). Nitric oxide modulates low-Mg2 + -induced epileptiform activity in rat hippocampal-entorhinal cortex slices. *Neurobiol. Dis.* 11 96–105. 10.1006/nbdi.2002.0533 12460549

[B148] Schuchmann S. Buchheim K. Meierkord H. Heinemann U. (1999). A relative energy failure is associated with low-Mg2 + but not with 4-aminopyridine induced seizure-like events in entorhinal cortex. *J. Neurophysiol.* 81 399–403. 10.1152/jn.1999.81.1.399 9914300

[B149] Schuchmann S. Kovács R. Kann O. Heinemann U. Buchheim K. (2001). Monitoring NAD(P)H autofluorescence to assess mitochondrial metabolic functions in rat hippocampal-entorhinal cortex slices. *Brain Res. Brain Res. Protoc.* 7 267–276. 10.1016/S1385-299X(01)00080-0 11431129

[B150] Schulz S. B. Klaft Z. J. Rösler A. R. Heinemann U. Gerevich Z. (2012). Purinergic P2X, P2Y and adenosine receptors differentially modulate hippocampal gamma oscillations. *Neuropharmacology* 62 914–924. 10.1016/j.neuropharm.2011.09.024 22001427

[B151] Shao L. R. Stafstrom C. E. (2017). Glycolytic inhibition by 2-deoxy-d-glucose abolishes both neuronal and network bursts in an in vitro seizure model. *J. Neurophysiol.* 118 103–113. 10.1152/jn.00100.2017 28404824 PMC5494374

[B152] Sinning A. Hübner C. A. (2013). Minireview: pH and synaptic transmission. *FEBS Lett.* 587 1923–1928. 10.1016/j.febslet.2013.04.045 23669358

[B153] Sotelo-Hitschfeld T. Niemeyer M. I. Mächler P. Ruminot I. Lerchundi R. Wyss M. T. (2015). Channel-mediated lactate release by K^+^-stimulated astrocytes. *J. Neurosci.* 35 4168–4178. 10.1523/JNEUROSCI.5036-14.2015 25762664 PMC6605297

[B154] Stafstrom C. E. Ockuly J. C. Murphree L. Valley M. T. Roopra A. Sutula T. P. (2009). Anticonvulsant and antiepileptic actions of 2-deoxy-D-glucose in epilepsy models. *Ann. Neurol.* 65 435–447. 10.1002/ana.21603 19399874 PMC2910719

[B155] Stafstrom C. E. Roopra A. Sutula T. P. (2008). Seizure suppression via glycolysis inhibition with 2-deoxy-D-glucose (2DG). *Epilepsia* 49(Suppl. 8), 97–100. 10.1111/j.1528-1167.2008.01848.x 19049601

[B156] Streijger F. Scheenen W. J. van Luijtelaar G. Oerlemans F. Wieringa B. Van der Zee C. E. (2010). Complete brain-type creatine kinase deficiency in mice blocks seizure activity and affects intracellular calcium kinetics. *Epilepsia* 51 79–88. 10.1111/j.1528-1167.2009.02182.x 19624717

[B157] Suh M. Bahar S. Mehta A. D. Schwartz T. H. (2005). Temporal dependence in uncoupling of blood volume and oxygenation during interictal epileptiform events in rat neocortex. *J. Neurosci.* 25 68–77. 10.1523/JNEUROSCI.2823-04.2005 15634768 PMC6725204

[B158] Szewczyk A. Skalska J. Głab M. Kulawiak B. Malińska D. Koszela-Piotrowska I. (2006). Mitochondrial potassium channels: from pharmacology to function. *Biochim. Biophys. Acta* 1757 715–720. 10.1016/j.bbabio.2006.05.002 16787636

[B159] Szybala C. Pritchard E. M. Lusardi T. A. Li T. Wilz A. Kaplan D. L. (2009). Antiepileptic effects of silk-polymer based adenosine release in kindled rats. *Exp. Neurol.* 219 126–135. 10.1016/j.expneurol.2009.05.018 19460372 PMC2728789

[B160] Takano T. Tian G. F. Peng W. Lou N. Lovatt D. Hansen A. J. (2007). Cortical spreading depression causes and coincides with tissue hypoxia. *Nat. Neurosci.* 10 754–762. 10.1038/nn1902 17468748

[B161] Tang C. M. Dichter M. Morad M. (1990). Modulation of the N-methyl-D-aspartate channel by extracellular H +. *Proc. Natl. Acad. Sci. U.S.A.* 87 6445–6449. 10.1073/pnas.87.16.6445 1696732 PMC54551

[B162] Thomsen K. Piilgaard H. Gjedde A. Bonvento G. Lauritzen M. (2009). Principal cell spiking, postsynaptic excitation, and oxygen consumption in the rat cerebellar cortex. *J. Neurophysiol.* 102 1503–1512. 10.1152/jn.00289.2009 19571198

[B163] Thornton R. C. Rodionov R. Laufs H. Vulliemoz S. Vaudano A. Carmichael D. (2010). Imaging haemodynamic changes related to seizures: comparison of EEG-based general linear model, independent component analysis of fMRI and intracranial EEG. *Neuroimage* 53 196–205. 10.1016/j.neuroimage.2010.05.064 20570736

[B164] Tolner E. A. Hochman D. W. Hassinen P. Otáhal J. Gaily E. Haglund M. M. (2011). Five percent CO2 is a potent, fast-acting inhalation anticonvulsant. *Epilepsia* 52 104–114. 10.1111/j.1528-1167.2010.02731.x 20887367 PMC3017646

[B165] Trussell L. O. Jackson M. B. (1987). Dependence of an adenosine-activated potassium current on a GTP-binding protein in mammalian central neurons. *J. Neurosci.* 7 3306–3316. 10.1523/JNEUROSCI.07-10-03306.1987 2822865 PMC6569168

[B166] Tyvaert L. LeVan P. Dubeau F. Gotman J. (2009). Noninvasive dynamic imaging of seizures in epileptic patients. *Hum. Brain Mapp.* 30 3993–4011. 10.1002/hbm.20824 19507156 PMC3767605

[B167] Urban A. Golgher L. Brunner C. Gdalyahu A. Har-Gil H. Kain D. (2017). Understanding the neurovascular unit at multiple scales: advantages and limitations of multi-photon and functional ultrasound imaging. *Adv. Drug Deliv. Rev.* 119 73–100. 10.1016/j.addr.2017.07.018 28778714

[B168] Van Gompel J. J. Bower M. R. Worrell G. A. Stead M. Chang S. Y. Goerss S. J. (2014). Increased cortical extracellular adenosine correlates with seizure termination. *Epilepsia* 55 233–244. 10.1111/epi.12511 24483230 PMC4104491

[B169] Vielhaber S. Von Oertzen J. H. Kudin A. F. Schoenfeld A. Menzel C. Biersack H. J. (2003). Correlation of hippocampal glucose oxidation capacity and interictal FDG-PET in temporal lobe epilepsy. *Epilepsia* 44 193–199. 10.1046/j.1528-1157.2003.38102.x 12558573

[B170] Wei A. C. Aon M. A. O’Rourke B. Winslow R. L. Cortassa S. (2011). Mitochondrial energetics, pH regulation, and ion dynamics: a computational-experimental approach. *Biophys. J.* 100 2894–2903. 10.1016/j.bpj.2011.05.027 21689522 PMC3123977

[B171] Wemmie J. A. Taugher R. J. Kreple C. J. (2013). Acid-sensing ion channels in pain and disease. *Nat. Rev. Neurosci.* 14 461–471. 10.1038/nrn3529 23783197 PMC4307015

[B172] Winkler E. A. Bell R. D. Zlokovic B. V. (2011). Central nervous system pericytes in health and disease. *Nat. Neurosci.* 14 1398–1405. 10.1038/nn.2946 22030551 PMC4020628

[B173] Xiong Z. Q. Saggau P. Stringer J. L. (2000). Activity-dependent intracellular acidification correlates with the duration of seizure activity. *J. Neurosci.* 20 1290–1296. 10.1523/JNEUROSCI.20-04-01290.2000 10662818 PMC6772378

[B174] Yaseen M. A. Sutin J. Wu W. Fu B. Uhlirova H. Devor A. (2017). Fluorescence lifetime microscopy of NADH distinguishes alterations in cerebral metabolism in vivo. *Biomed. Opt. Express.* 8 2368–2385. 10.1364/BOE.8.002368 28663879 PMC5480486

[B175] Yemisci M. Gursoy-Ozdemir Y. Vural A. Can A. Topalkara K. Dalkara T. (2009). Pericyte contraction induced by oxidative-nitrative stress impairs capillary reflow despite successful opening of an occluded cerebral artery. *Nat. Med.* 15 1031–1037. 10.1038/nm.2022 19718040

[B176] Young D. Dragunow M. (1994). Status epilepticus may be caused by loss of adenosine anticonvulsant mechanisms. *Neuroscience* 58 245–261. 10.1016/0306-4522(94)90032-9 8152537

[B177] Zhao M. Ma H. Suh M. Schwartz T. H. (2009). Spatiotemporal dynamics of perfusion and oximetry during ictal discharges in the rat neocortex. *J. Neurosci.* 29 2814–2823. 10.1523/JNEUROSCI.4667-08.2009 19261877 PMC2745405

[B178] Zhao M. Nguyen J. Ma H. Nishimura N. Schaffer C. B. Schwartz T. H. (2011). Preictal and ictal neurovascular and metabolic coupling surrounding a seizure focus. *J. Neurosci.* 31 13292–13300. 10.1523/JNEUROSCI.2597-11.2011 21917812 PMC3191875

[B179] Ziemann A. E. Schnizler M. K. Albert G. W. Severson M. A. Howard M. A. III Welsh M. J. (2008). Seizure termination by acidosis depends on ASIC1a. *Nat. Neurosci.* 11 816–822. 10.1038/nn.2132 18536711 PMC2553357

[B180] Zonta M. Angulo M. C. Gobbo S. Rosengarten B. Hossmann K. A. Pozzan T. (2003). Neuron-to-astrocyte signaling is central to the dynamic control of brain microcirculation. *Nat. Neurosci.* 6 43–50. 10.1038/nn980 12469126

[B181] Zsurka G. Kunz W. S. (2015). Mitochondrial dysfunction and seizures: the neuronal energy crisis. *Lancet Neurol.* 14 956–966. 10.1016/S1474-4422(15)00148-9 26293567

[B182] Zubler F. Steimer A. Gast H. Schindler K. A. (2014). Seizure termination. *Int. Rev. Neurobiol.* 114 187–207. 10.1016/B978-0-12-418693-4.00008-X 25078503

